# The burden of influenza‐associated respiratory hospitalizations in Bhutan, 2015‐2016

**DOI:** 10.1111/irv.12605

**Published:** 2018-10-23

**Authors:** Binay Thapa, Katherine Roguski, Eduardo Azziz‐Baumgartner, Karen Siener, Philip Gould, Thinley Jamtsho, Sonam Wangchuk

**Affiliations:** ^1^ Royal Centre for Disease Control Ministry of Health Thimphu Bhutan; ^2^ Influenza Division Centers for Disease Control and Prevention Atlanta Georgia; ^3^ Regional Office for South East Asia World Health Organization New Delhi India

**Keywords:** Bhutan, burden, influenza, severe acute respiratory infections, surveillance

## Abstract

**Background:**

Influenza burden estimates help provide evidence to support influenza prevention and control programs. In this study, we estimated influenza‐associated respiratory hospitalization rates in Bhutan, a country considering influenza vaccine introduction.

**Methods:**

Using real‐time reverse transcription‐polymerase chain reaction laboratory results from severe acute respiratory infection (SARI) surveillance, we estimated the proportion of respiratory hospitalizations attributable to influenza each month among patients aged <5, 5‐49, and ≥50 years in six Bhutanese districts for 2015 and 2016. We divided the sum of the monthly influenza‐attributed hospitalizations by the total of the six district populations to generate age‐specific rates for each year.

**Results:**

In 2015, 10% of SARI patients tested positive for influenza (64/659) and 18% tested positive (129/736) in 2016. The incidence of influenza‐associated hospitalizations among all age groups was 50/100 000 persons (95% confidence interval [CI]: 45‐55) in 2015 and 118/100 000 persons (95% CI: 110‐127) in 2016. The highest rates were among children <5 years: 182/100 000 (95% CI: 153‐210) in 2015 and 532/100 000 (95% CI: 473‐591) in 2016. The second highest influenza‐associated hospitalization rates were among adults ≥50 years: 110/100 000 (95% CI: 91‐130) in 2015 and 193/100 000 (95% CI: 165‐221) in 2016.

**Conclusions:**

Influenza viruses were associated with a substantial burden of severe illness requiring hospitalization especially among children and older adults. These findings can be used to understand the potential impact of seasonal influenza vaccination in these age groups.

## 1 INTRODUCTION

Each year, influenza virus infections are a major contributor to hospitalizations worldwide.^1^ Many high‐ and upper‐middle‐income countries have used influenza‐associated burden estimates to identify target groups for influenza prevention and control and to explore the cost‐effectiveness of these interventions.^2^, ^3^, ^4^, ^5^, ^6^ Despite increasing global influenza surveillance capacity and an improved understanding of influenza virus seasonality and epidemiology, estimates of influenza‐associated burden are limited in low‐ and middle‐income countries.^1^, ^7^ Country‐specific influenza‐associated burden estimates generated through the use of viral surveillance data can provide useful information to ministries of health (MOH) and national immunization technical advisory groups (NITAG) as they seek to understand the potential value of introducing seasonal influenza vaccines in their country.^3^, ^8^

**Figure 1:**
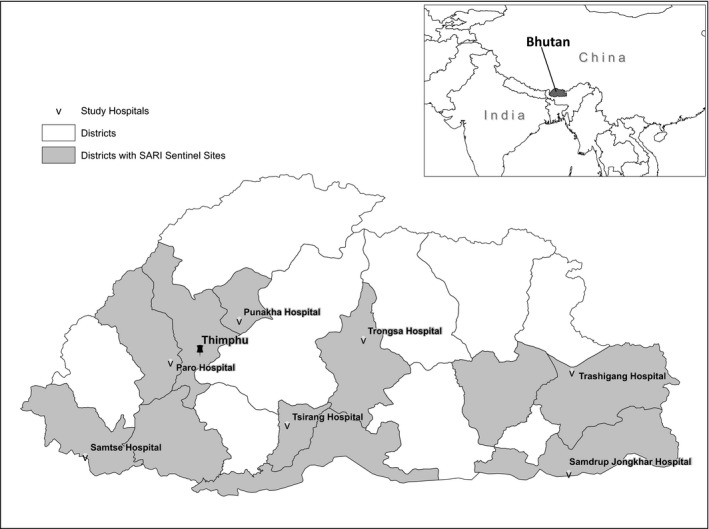
Districts in Bhutan where surveillance for severe acute respiratory infections (SARI) was conducted (gray shading) and location of seven district‐level hospitals evaluated for this analysis

The Kingdom of Bhutan is a lower‐middle‐income country located in Eastern Himalaya between China to the north and India to the east, west, and south, with a population of roughly 760 000 (Figure 1).^9^ Climate across Bhutan varies based on changes in elevation, from high Himalayan peaks in the north to the tropical plains along the Indian border. The government of Bhutan has reported sporadic outbreaks of A(H5N1) virus in domestic poultry from 2010 to 2016, mainly in districts near the Indian border.^10^ The majority of Bhutan's population lives in rural settings with rugged terrain making cross country travel challenging and access to healthcare services difficult.^11^ The government of Bhutan provides all healthcare services, including medications and routine immunizations, free of charge to its citizens. While seasonal influenza is not part of Bhutan's routine immunization schedule, the government of Bhutan is exploring the utility of its introduction among WHO recommended target groups.^12^ In addition, Bhutanese referral hospitals also have a stockpile of antivirals from WHO but have not yet identified priority groups to receive this medication during seasonal epidemics.

In 2008, the Royal Centre for Disease Control (RCDC), MOH in Bhutan, in conjunction with the United States Armed Force Research Institute for Medical Sciences, initiated sentinel surveillance for influenza‐like illness (ILI) in outpatients.^13^ In 2012, RCDC expanded surveillance to hospitalized patients with severe acute respiratory infections (SARI) in collaboration with the US Centers for Disease Control and Prevention (CDC). Royal Centre for Disease Control selected 11 hospital sites (seven district and four referral hospitals) to participate in the SARI surveillance network based on their geographical, climatic, and demographic representativeness. The hospitals participating in influenza surveillance represent 11 of Bhutan's 20 districts, all three of the country's geographic regions, and 35% of all hospitals in the country (total = 31). Data from this influenza surveillance platform can be used to estimate influenza‐associated hospitalization rates and inform an investment case for influenza vaccination and control measures.^14^

In this analysis, we used SARI viral surveillance data, nationally reported respiratory coded hospital discharge data, and census data to estimate age group‐specific influenza‐associated respiratory hospitalizations. These findings are intended to help the Bhutanese MOH and NITAG assess the value of influenza vaccination among children and older adults (two of the WHO recommended target groups), the targeting of empiric antiviral use among hospitalized patients during the influenza season, and other influenza prevention and control measures.^12^

## 2 METHODS

### 2.1 Design

We used influenza viral laboratory results from nationally representative SARI surveillance to estimate the proportion of respiratory hospital discharges in six districts attributable to influenza each month for 2015 and 2016; the approach used methods adapted from the WHO *Manual for Estimating Disease Burden Associated with Seasonal Influenza*.^14^ We then summed estimates of influenza‐associated hospitalization across each of the years and district by age group and divided the sum by the age‐specific census population to generate influenza‐associated hospitalization rates among persons aged <5, 5‐49, and ≥50 years. We first focused on seven of the 11 SARI sentinel sites that were district‐level hospitals with a defined catchment population, which was the known population of persons residing around a hospital that seek care at that particular hospital (Figure 1). After evaluating the SARI surveillance and respiratory discharge data, we excluded one district hospital because of the poor concurrence of discharge diagnosis data in hospital records verses what the hospital reported to the national office. The four remaining sentinel hospitals were referral hospitals, without defined catchment populations; although we incorporated their virological surveillance data into this analysis, we did not generate influenza‐associated hospitalization rates specifically for these sites.

### 2.2 SARI surveillance network

During 2015‐2016, a SARI case‐patient in Bhutan was defined as any person hospitalized with an acute respiratory infection with: (a) history of fever or measured fever ≥38°C, (b) cough or sore throat, (c) and symptom onset in the last 10 days. Surveillance nurses sought to enroll and collect specimens from all hospitalized patients meeting the SARI case definition each day at the 11 sentinel hospitals (Figure 1). After obtaining informed consent and clinical and epidemiological information on standardized forms, nurses collected throat swabs from all enrolled case‐patients. Sentinel sites submitted patient information and the total number of SARI case‐patients weekly through a web‐based influenza surveillance system. Specimens were stored in viral transport media tubes at 2‐8°C and shipped weekly to RCDC in Thimphu in a cold box.

Laboratory staff at RCDC tested all specimens by real‐time reverse transcription‐polymerase chain reaction for the presence of influenza A and B viruses using methods adapted from CDC protocols.^15^ Specimens positive for influenza A were further tested for influenza A virus subtypes. In addition to testing for seasonal virus subtypes, patients with recent exposure to avian influenza outbreaks among poultry or with recent international travel history were also tested for influenza A(H5) or A(H7) viruses, respectively.

### 2.3 Evaluation of SARI case‐patient identification

In order to determine whether the number of SARI case‐patients reported to RCDC accurately reflected the totality of patients meeting the SARI case definition, we conducted a retrospective medical chart review at four of the seven district‐level SARI sentinel site hospitals, which we selected for their accessibility. At each hospital, we reviewed approximately 100 hospitalized patient medical charts from three to eight randomly selected weeks during 2015 and collected demographic, symptom, and discharge information. As annual hospital admissions varied widely between these four hospitals, the number of randomly selected weeks varied by hospital in order to review a similar number of charts at each hospital. We classified each patient according to the Bhutanese SARI case definition based on recorded symptom data. We then compared the number of SARI case‐patients identified during the chart review to the number of SARI case‐patients reported to RCDC during the same time period.

### 2.4 Observed respiratory hospitalizations

At hospitals throughout Bhutan, records staff coded the primary discharge diagnosis for each hospitalization using the 10th revision of the International Statistical Classification of Diseases and Related Health Problems (ICD‐10) and submitted monthly summary counts to the Health Information Management System Unit in the MOH quarterly. We obtained counts of all primary respiratory disease discharges (J00‐J99) by age group, month, and hospital for 2015 and 2016 from the MOH.

### 2.5 Evaluation of ICD‐10 coded hospitalizations

In all seven SARI sentinel sites that are district‐level hospitals, we additionally conducted a retrospective hospital‐based logbook review to validate the number of respiratory ICD‐10 coded hospitalizations reported to the MOH each month. We conducted this evaluation to validate data in the national MOH database before it was included in the pooled analysis. At each hospital, we reviewed discharge diagnoses (both free text and ICD‐10 coded) in hospital logbooks in randomly selected months during 2015‐2016. We recorded demographic information (age and district of residence) and primary discharge diagnosis information (free text or ICD‐10 coded) for every hospitalized patient with a reported respiratory discharge diagnosis. We summarized the number of respiratory hospitalizations by month, age group, and hospital and compared it to the number of respiratory hospitalizations reported to the MOH national hospitalization database for the same month.

### 2.6 Calculation of influenza‐associated respiratory hospitalizations

After excluding one SARI sentinel site following the data evaluations, we estimated annual influenza‐associated respiratory hospitalizations for the six remaining districts in Bhutan where the SARI surveillance sentinel site was the only admitting hospital within that district. These six districts represented 31% of the national population. We excluded Hospital A because the number of respiratory diagnoses reported to the MOH and the number recorded in the hospital logbooks were so divergent. We calculated the monthly proportion of specimens testing positive for influenza virus across all age groups and sentinel sites for 2015‐2016. We were not able to stratify these data by age group or region because of the low number of influenza virus‐positive specimens identified each year. We then estimated influenza‐associated respiratory hospitalizations in each district using the following formula:

$$H_{a,d}=\sum_{m}N_{m,a,d}\;\times \;\frac{I_{m}}{T_{m}}$$

where:

*H*_a,d_: influenza‐associated respiratory hospitalizations for age group (*a*) and district (*d*),

*N*_m,a,d_: number of respiratory ICD‐10 coded hospitalizations from the national database for month (m), age group (*a*), and district (*d*),

*I*_m_: national number of influenza‐positive SARI specimens for month (m),

*T*_m_: national number of SARI specimens tested for influenza viruses for month (m).

We next summed estimates of influenza‐associated respiratory hospitalizations across all six districts. We assumed the catchment population of each of the hospitals was the district population, as all health centers referred severely ill patients to their respective district hospitals and no other hospitals were located in these districts. We obtained mid‐year district‐level population estimates for 2015 by age groups from the Bhutanese National Statistics Bureau^9^ and calculated pooled rates by dividing the sum of influenza‐associated hospitalizations across the six sites by the sum of all six district populations for 2015 and 2016. We extrapolated these combined rates to the national level using national population estimates to generate national estimates of influenza‐associated respiratory hospitalizations. We calculated the confidence intervals assuming a binomial distribution for the variance in the proportion of samples testing positive for influenza and a Poisson distribution for the variance in respiratory hospitalizations using the following formula:

$$\begin{array}{cc} 95\%\;{\text{CI}}_{a}= & H_{a}\;\pm 1.96\;\\ & \times \;\sqrt{\sum_{m}\left(N_{a,m}\times \frac{(P_{m}\times \left(1-P_{m}\right)}{T_{m}}\right)+\left(N_{a,m}\times P_{m}^{2}\right)+\left(\frac{(P_{m}\times \left(1-P_{m}\right)}{T_{m}}\times N_{a,m}^{2}\right)} \end{array}$$

where:

95% CI_a_: 95% confidence interval of influenza‐associated respiratory hospitalizations for age group (a);

*H*_a_: influenza‐associated respiratory hospitalizations for age group (*a*);

*N*_a,m_: number of respiratory hospitalizations for age group (*a*) and month (m);

*P*_m_: national proportion of influenza‐positive specimens for month (m);

*T*_m_: national number of SARI specimens tested for influenza viruses for month (m).

### 2.7 Ethics statement

This project was part of an influenza control program evaluation; only anonymized surveillance data were used for this analysis.

## 3 RESULTS

### 3.1 SARI surveillance

**Figure 2:**
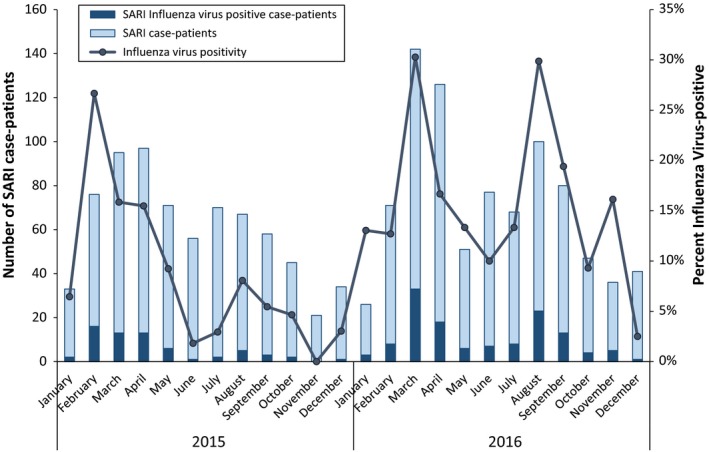
Number of SARI case‐patients and influenza virus‐positive case‐patients by month, Bhutan, 2015‐2016

During January 2015 to December 2016, surveillance staff across the 11 participating SARI sentinel sites collected specimens from 1395 hospitalized patients meeting the SARI case definition. The median number of days from symptom onset to specimen collection was four (interquartile range: 2‐6 days). The majority (61%) of the 1395 case‐patients were children aged <5 years; 21% (n = 292) were <1 year; and 40% (n = 555) were aged 1‐4 years (Table 1). Seventy‐five percent (1020/1361) of enrolled SARI case‐patients reported trouble breathing, and 73% (997/1361) of enrolled SARI case‐patients were diagnosed with pneumonia. Sixty‐four (9.7%) case‐patients in 2015 and 129 (17.5%) in 2016 had influenza virus‐positive respiratory samples. Among the influenza virus‐positive specimens, the majority (64%) were positive for influenza A(H1N1)pdm09 virus in 2015, and in 2016, the majority (56%) were positive for influenza B viruses. Peaks in influenza virus detection occurred from February to April both years (66% and 46% of total influenza‐positive samples in 2015 and 2016, respectively), and a secondary peak was seen from July to September in 2016 (34% of total influenza‐positive samples) (Figure 2). No SARI patients tested positive for influenza A(H5) or A(H7) viruses in 2015‐2016.

**Table 1:** Demographic characteristics of hospitalized patients meeting the severe acute respiratory illness (SARI) case definition and influenza virus confirmation, Bhutan, 2015‐2016

Characteristics	Number of SARI case‐patients, N (%)	
	2015 (N = 659)	2016 (N = 736)
Age		
<1 y	108 (16)	184 (25)
1‐<5 y	309 (47)	248 (34)
5‐<15 y	78 (12)	84 (11)
15‐<30 y	59 (9)	90 (12)
30‐<50 y	50 (8)	44 (6)
≥50 y	55 (8)	73 (10)
Age missing	0 (0)	13 (2)
Reported difficulty breathing	457 (73)^a^	563 (76)
Diagnosed with pneumonia	461 (74)^a^	536 (73)
Laboratory‐confirmed influenza	64 (10)	129 (18)
Influenza A (H1N1)pdm09	41 (6)	12 (2)
Influenza A (H3N2)	15 (2)	36 (5)
Influenza A (not subtyped)	0 (0)	9 (1)
Influenza B	8 (1)	72 (10)

a Information on signs and symptoms missing for 34 SARI case‐patients in 2015.

### 3.2 Evaluation of SARI case‐patient identification

In hospitals where the study team evaluated SARI case‐patient identification, the combined number of SARI case‐patients identified and reported by hospital staff through routine surveillance during the review period (N = 14) represented 36% (95% CI: 21‐51) of those identified through our medical chart review (N = 39) (Table S1). Each week, however, the number of SARI case‐patients identified in medical charts was very small; in 10 (56%) of the 18 weeks reviewed, there was ≤1 SARI case‐patient identified in the medical charts or reported through the surveillance system.

### 3.3 Respiratory hospitalizations

Hospitals across Bhutan reported 11 782 respiratory coded (J00‐J99) hospital discharge diagnoses to the MOH in 2015 and 13 697 in 2016. The six district hospitals used in this analysis reported 12% (3138/25 479) of the nationally reported respiratory hospitalizations. Forty‐five percent (n = 1421) of the 3138 respiratory hospitalizations in these six hospitals were children aged <5 years, 26% (n = 829) were persons aged 5‐49 years, and 28% (n = 888) were adults aged ≥50 years. Our review of the discharge logbooks identified differences between what was recorded in logbooks at the seven district hospitals where we conducted our evaluation and what was reported nationally (Table S2). On average, these differences were small (0.1%‐12.8% fewer discharges recorded in national datasets than recorded in the hospital logbooks). In Hospital A, however, there was a 7‐fold difference in what was recorded in the hospital logbook vs the national database.

### 3.4 Influenza‐associated respiratory hospitalizations

Across the six sentinel sites, we estimated 116 (95% CI: 104‐127) influenza‐associated respiratory hospitalizations occurred across all ages in 2015 and 276 (95% CI: 256‐296) in 2016 (Table 2). These equated to an all age rate of 50 per 100 000 persons (95% CI: 45‐55) in 2015 and 118 per 100 000 persons (95% CI: 110‐127) in 2016. In both years, the highest rates were among children <5 years: 182 per 100 000 persons (95% CI: 153‐210) in 2015 and 532 per 100 000 persons (95% CI: 473‐591) in 2016. The second highest rates were among adults ≥50 years: 110 per 100 000 (95% CI: 91‐130) in 2015 and 193 per 100 000 (95% CI: 165‐221) in 2016. Rates were consistently higher across age groups in 2016 when influenza B virus was predominant compared to 2015 when influenza A(H1N1)pdm09 virus predominated. When we extrapolated these rates to the national population, we estimated 376 (95% CI: 339‐413) influenza‐associated respiratory hospitalizations among all ages occurred in 2015 and 896 (95% CI: 830‐962) in 2016 (Table 3).

**Table 2:** Pooled numbers of respiratory hospitalizations, estimates of influenza‐associated hospitalizations, and influenza‐associated hospitalization rates (per 100 000) from six districts in Bhutan, 2015‐2016

Age groups	Respiratory hospitalizations N (95% CI)	Influenza‐associated hospitalization estimates N (95% CI)	Population, six districts	Influenza‐associated hospitalization rates (per 100 000) (95% CI)
2015				
<5 y	590 (542‐638)	46 (39‐54)	25 453	182 (153‐210)
5‐49 y	387 (348‐426)	31 (26‐37)	172 882	18 (15‐21)
≥50 y	463 (421‐505)	38 (32‐45)	34 887	110 (91‐130)
All Age	1440 (1366‐1514)	116 (104‐127)	233 222	50 (45‐55)
2016				
<5 y	831 (774‐888)	135 (120‐151)	25 453	532 (473‐591)
5‐49 y	442 (401‐483)	73 (63‐83)	172 882	42 (37‐48)
≥50 y	425 (385‐465)	67 (58‐77)	34 887	193 (165‐221)
All Age	1698 (1617‐1779)	276 (256‐296)	233 222	118 (110‐127)

**Table 3:** National influenza‐associated respiratory hospitalization estimates for Bhutan extrapolated from the pooled rate from six districts, 2015‐2016

Age groups	National census population (2015)	Influenza‐associated hospitalization estimates, N (95% CI)	
		2015	2016
<5 y	82 716	150 (126‐174)	440 (391‐489)
5‐49 y	566 291	102 (84‐120)	240 (208‐272)
≥50 y	108 035	119 (98‐140)	209 (179‐239)
All Age	757 042	376 (339‐413)	896 (830‐962)

## 4 DISCUSSION

We observed a high burden of seasonal influenza virus infections among respiratory hospitalizations in 2015‐2016 across Bhutan, especially among children <5 years and older adults. As seasonal influenza vaccine is not currently available in Bhutan, the Bhutanese MOH could use these burden estimates to assess the potential value of seasonal vaccine introduction and other influenza control measures. For example, the Bhutanese MOH could use these hospitalization rates to explore seasonal influenza vaccine program cost‐effectiveness and cost benefit or to determine priority groups to receive stockpiled antivirals during seasonal epidemics. The Bhutanese MOH is also updating its pandemic plan, and these influenza‐associated hospitalization rates might help them better plan for the number of vaccine and antiviral doses needed during an influenza pandemic.

Our estimates varied substantially between 2015 and 2016, with a greater than twofold difference in the all age burden between the 2015 and 2016 seasons. Such seasonal variability in influenza hospitalization burden has been observed in other countries^2^, ^16^, ^17^ and may be due to differences in influenza virus circulation and host susceptibility.^18^, ^19^ The higher proportion of samples testing positive for influenza viruses we observed in the SARI surveillance data in 2016 compared to 2015, especially for the secondary peak from July‐September 2016, was consistent with trends we observed in the national Bhutanese outpatient ILI surveillance data during the same seasons.^20^ This variability between seasonal estimates highlights the importance of generating influenza disease burden estimates over more than one season. The RCDC could consider updating their estimates of influenza disease burden with additional years of data in the future in order to understand this variability over a longer time series.

Our all age rate estimates were similar to previously published estimates from neighboring countries where seasonal influenza vaccine use was low, such as rural India, central China, and Thailand.^17^, ^21^, ^22^ The greatest influenza‐associated hospitalization burden we observed was among children <5 years of age, which is consistent with other published studies from low‐ and middle‐income countries such as Bangladesh,^23^ Egypt,^24^ the Philippines,^25^ and South Africa.^26^ Our estimate from 2015 for children <5 years (182 [95% CI: 153‐210] per 100 000) was also similar to pooled estimates published for children <5 years from developing nations (150 [95% CI: 105‐216] per 100 000) and for the South East Asia WHO region (157 [95% CI: 76‐326] per 100 000), of which Bhutan is a member.^1^

Our evaluation of the SARI surveillance platform in Bhutan identified significant underreporting of SARI case‐patients, which led us to use respiratory ICD‐10 coded hospital discharges as a proxy for SARI in our influenza‐associated hospitalization rate estimates. While many developing countries have strengthened influenza surveillance since 2005^7^ and may want to leverage surveillance data to generate disease burden estimates, our findings suggest the value of evaluating surveillance data before using it for secondary analyses. Following our evaluation, RCDC staff conducted retraining on SARI case‐patient identification and surveillance reporting for clinicians participating in the SARI surveillance system in hopes of strengthening the Bhutanese surveillance system.

Our evaluation of ICD‐10 coded hospitalization data identified relatively minor discrepancies between what was recorded in hospital logbooks compared to national databases for all but one hospital. These differences prompted us to drop Hospital A from the analysis. After we completed this study, we determined that Hospital A had reported the combined number of respiratory hospitalizations and emergency room visits to the national office rather than just hospitalization inadvertently inflating their numbers. This evaluation demonstrated that while using the national level databases may be expedient, national level data should also be validated. Evaluating the nationally reported hospitalization data from our sentinel sites made us more confident in the pooled estimate.

We acknowledge important limitations to our influenza‐associated hospitalization rates. First, our study attributed influenza laboratory findings to respiratory hospitalizations in six districts based on the results of sampled SARI case‐patients of all ages identified throughout the national surveillance system. The number of influenza hospitalizations would likely be different if all hospitalized patients had been tested for influenza; however, the age distributions of sampled SARI case‐patients and reported respiratory hospitalizations were similar, which might minimize this difference.^27^, ^28^ Second, hospitals in Bhutan only reported one discharge diagnosis, rather than all underlying or secondary diagnoses, which likely underestimated the true number of hospitalizations for respiratory illnesses. Third, we extrapolated rates from six districts that did not include the capital, Bhutan's largest city, to all of Bhutan; these districts may not be representative of all of Bhutan. Fourth, we did not include any districts with referral hospitals, which may underestimate severe respiratory disease in the country. Last, while some residents in one district might have sought medical care in another district, we assumed district‐level populations were a fair approximation of each district hospital catchment population because of the remoteness and relative isolation of Bhutanese communities.

## 5 CONCLUSION

Our findings suggest that each year hundreds of influenza‐associated respiratory hospitalizations occur throughout Bhutan, especially among young children and older adults. These findings demonstrate the magnitude of seasonal influenza burden among different age groups and could be used to explore the potential value of introducing influenza prevention and control measures. For example, these estimates could be used to estimate the number of influenza cases that could be averted through an influenza vaccine program, information that would be valuable for the Bhutanese MOH and NITAG when considering seasonal influenza vaccine introduction.

## DISCLAIMER

The findings and conclusions in this report are those of the author(s) and do not necessarily represent the official position of the US Centers for Disease Control and Prevention or the World Health Organization.

## CONFLICT OF INTEREST

Co authors have no conflict of interests to declare.
