# Supplementary material for: The burden of influenza‐associated respiratory hospitalizations in Bhutan, 2015‐2016
Source: Influenza Other Respir Viruses. 2018 Oct 23;13(1):28–35. doi: 10.1111/irv.12605 (PMC6304319; doi:10.1111/irv.12605)
Supplement: Supplementary file 1 [file IRV-13-28-s001.docx]

*The Burden of Influenza-Associated Respiratory Hospitalizations in Bhutan, 2015-2016*

Binay Thapa, Katherine Roguski, Eduardo Azziz-Baumgartner, Karen Siener, Philip Gould, Thinley Jamtsho, and Sonam Wangchuk

Supplementary Material:

**Supplemental Table 1.** Comparison of number of SARI case-patients identified through a retrospective chart review to the number of SARI case-patients reported to RCDC during the same time during randomly selected weeks for four district hospitals, 2015.

| **Hospital** | **Weeks Reviewed*** | **Number of SARI Case-patients Identified by Chart Review** | **Number of SARI Case-patients Reported** | **% SARI Reported** | **Weekly Range of % SARI Reported** |
| --- | --- | --- | --- | --- | --- |
| A | 5, 34, 49 | 2 | 1 | 50 | 0 – 0 |
| B | 8, 19, 31 | 8 | 3 | 38 | 0 – 75 |
| C | 6, 11, 20, 23, 29, 33, 41, 46 | 9 | 3 | 33 | 0 – 100 |
| D | 9, 20, 36, 50 | 20 | 7 | 35 | 0 – 67 |
| **Summary** | | **39** | **14** | **36 (95% CI: 21** – **51)** | |

*Week number corresponds to the US National Notifiable Disease Surveillance System numbering system for weeks in 2015.

**Supplemental Table 2.** Comparison of respiratory hospital admissions identified at each hospital to the number reported to the MOH for the same time during randomly selected months for seven district hospitals, 2015-2016.

| **Hospital** | **Months Reviewed** | **Respiratory Admissions Identified in Log Book** | **Respiratory Admissions Reported to MOH** | **Average of Monthly Difference Between Sources** | **Monthly Range of Differences Between Sources** |
| --- | --- | --- | --- | --- | --- |
| A | Feb, Jun, Aug, Dec 2015 | 92 | 705 | -153.3 | -122 – -185 |
| B | Feb, May, Aug, Nov 2015 | 117 | 66 | 12.8 | 8 – 16 |
| C | All 2015 | 144 | 143 | 0.1 | -2 – 2 |
| D | Mar, May, Sep, Dec 2015 | 127 | 126 | 0.3 | -4 – 3 |
| E | Mar, Jun, Aug, Nov 2015; Jan-Jun 2016 | 217 | 198 | 1.9 | -4 – 12 |
| F | Feb, Apr-May, Aug-Sep, Nov 2015; Mar, Jun 2016 | 215 | 156 | 7.4 | -5 – 47 |
| G | Feb-Mar, May, Aug, Dec 2015; Jan-Jun 2016 | 417 | 356 | 5.5 | -26 – 24 |
